# Accidental dexmedetomidine overdose in preterm newborns: a report of 3 cases

**DOI:** 10.1186/s13052-025-02060-1

**Published:** 2025-07-06

**Authors:** Giulia Paviotti, Mariabeatrice D’Agostini, Isabella Mauro, Federica Bortolotti, Rossella Gottardo, Carla Pittini

**Affiliations:** 1 Division of Neonatology, University Hospital of Udine, Via Pozzuolo 330, Udine, Italy; 2 Department of Medicine-DMed, University of Udine, Udine, Italy; 3 Department of Diagnostic and Public Health, Unit of Forensic Medicine, University of Verona, Verona, Italy

**Keywords:** Dexmedetomidine, Medication error, Newborn, Overdose, Case report

## Abstract

**Background:**

Dexmedetomidine use in neonatal units is increasing. Data on its safety are still limited. There are no previous reports of clinical presentation of dexmedetomidine overdose in newborns.

**Case presentation:**

Three babies simultaneously developed a similar clinical picture of recurrent apneas, a typical “gasping” breathing pattern, irritability followed by hypotonia and hyporeactivity, hyperglycaemia, hypocapnia, increase in lactates and a suppression-burst pattern on CFM/EEG. Babies required intubation and mechanical ventilation due to poor respiratory effort. Symptoms resolved completely in a few hours. Dexmedetomidine was administered enterally by a nasogastric tube in place of caffeine due to “look alike” medication error. Dexmedetomidine was retrieved in biological samples. Babies were developing regularly at post-discharge follow up visits.

**Conclusion:**

Dexmedetomidine overdose due to medication error is possible in newborns and should be suspected in case of clinical presentations similar to the one we reported. Measures should be implemented in neonatal units for a safe use of dexmedetomidine.

**Supplementary Information:**

The online version contains supplementary material available at 10.1186/s13052-025-02060-1.

## Background

The use of dexmedetomidine in neonatal intensive care units (NICUs) has increased significantly in recent years [1]. Dexmedetomidine, which is an alpha-2 adrenergic agonist [2], is employed to manage pain, anxiety, and agitation in premature and term infants undergoing invasive procedures, therapeutic hypothermia or requiring mechanical ventilation [3].

Unlike benzodiazepines or opioids, dexmedetomidine offers the advantage of maintaining respiratory drive during sedation. It also has minimal effects on haemodynamics and gastro-intestinal motility [3]. Moreover, preclinical data have shown potential anti-inflammatory and neuroprotective properties [4].

Although limited adverse effects (bradycardia, agitation, hypertension, hypotension, withdrawal) have been described in neonatal clinical studies [3, 5], evidence on dexmedetomidine safety in newborns is still scarce [6]. Moreover, pharmacokinetics is still poorly known in newborns, especially in those born preterm, and it might potentially differ substantially from that of later ages [5].

Intravenous dexmedetomidine overdose has been reported in both adults [7, 8] and children [9–13]. In contrast, to the best of our knowledge, no data on dexmedetomidine overdose in preterm newborns have been published so far.

The present paper reports three cases of dexmedetomidine overdose in preterm newborns following accidental intragastric administration due to “look alike” medication error. Moreover, it is detailed the management of the incident by the clinicians and hospital risk management.

## Case presentation

Clinical data were retrieved from medical records. Parents of the babies were informed and provided written informed consent.

These reports were written following the CARE guidelines [14] (see Supplementary Material, CARE-checklist_dex.pdf).

### Case 1

A baby girl was born after a monochorionic-diamniotic twin pregnancy at 34 + 6 weeks of gestational age from vaginal birth. Birth weight was 2170 g. Postnatal adaptation was good with 1–5 min Apgar scores of 8–9. She required CPAP for mild respiratory distress and apnoea, discontinued on the 4th day of life (DOL4). Enteral caffeine was started at 9 h of life and continued because apnoeic episodes were still occurring.

The baby was growing regularly on full enteral feeds by nasogastric tube and breathing in room air with stable vital signs when, on DOL9, at 36 + 1 weeks of postconceptional age, at 9:00 a.m. she suddenly started to show irritability followed by decreased reactivity, hypotonia and repeated frequent episodes of apnoea, an irregular breathing pattern with a “gasping” appearance (Supplementary Material, Video dex.MP4), a poorly variable heart rate of 120–125 beats/min, no hypotension.

Urgent blood tests showed hypocapnia (23 mmHg), lactates increase (6.1 mMol/L) and hyperglycemia (glucose 228 mg/dL); infection markers were negative (Table 1).

**Table 1:** Arterial blood test results

	Case 1	Case 2	Case 3
pH	7.44	7.64	7.46
pCO2 (mmHg)	23	16	26
pO2 (mmHg)	63	88	77
BEecf (mmol/L)	-6.1	-3.7	-5.3
HCO3- (mmol/L)	15.6	17.2	18.5
Lactate (mmol/L)	6.1	3.0	4.6
Glucose (mg/dL)	228	307	238
White blood cells/uL	9920	7300	10570
Procalcitonin (ng/mL)	0.10	0.09	0.16
CRP (mg/L)	< 0.6	< 0.6	< 0.6

Empirical antibiotic therapy and acyclovir were started. Molecular panels on nasopharyngeal secretions, blood, stool, and cerebral spinal fluid were negative. Head, lung and cardiac ultrasounds and ECG were regular.

Cerebral Function Monitoring (CFM), initiated 1 h after onset showed a suppression-burst pattern. Decreased respiratory effort required intubation and mechanical ventilation 3 h after symptoms onset.

Pending toxicological tests, a dose of both naloxone and flumazenil was given empirically, with no effect. In any case, urine toxicological tests resulted to be negative for opiates, benzodiazepines, and other drugs of abuse.

**Fig. 1:**
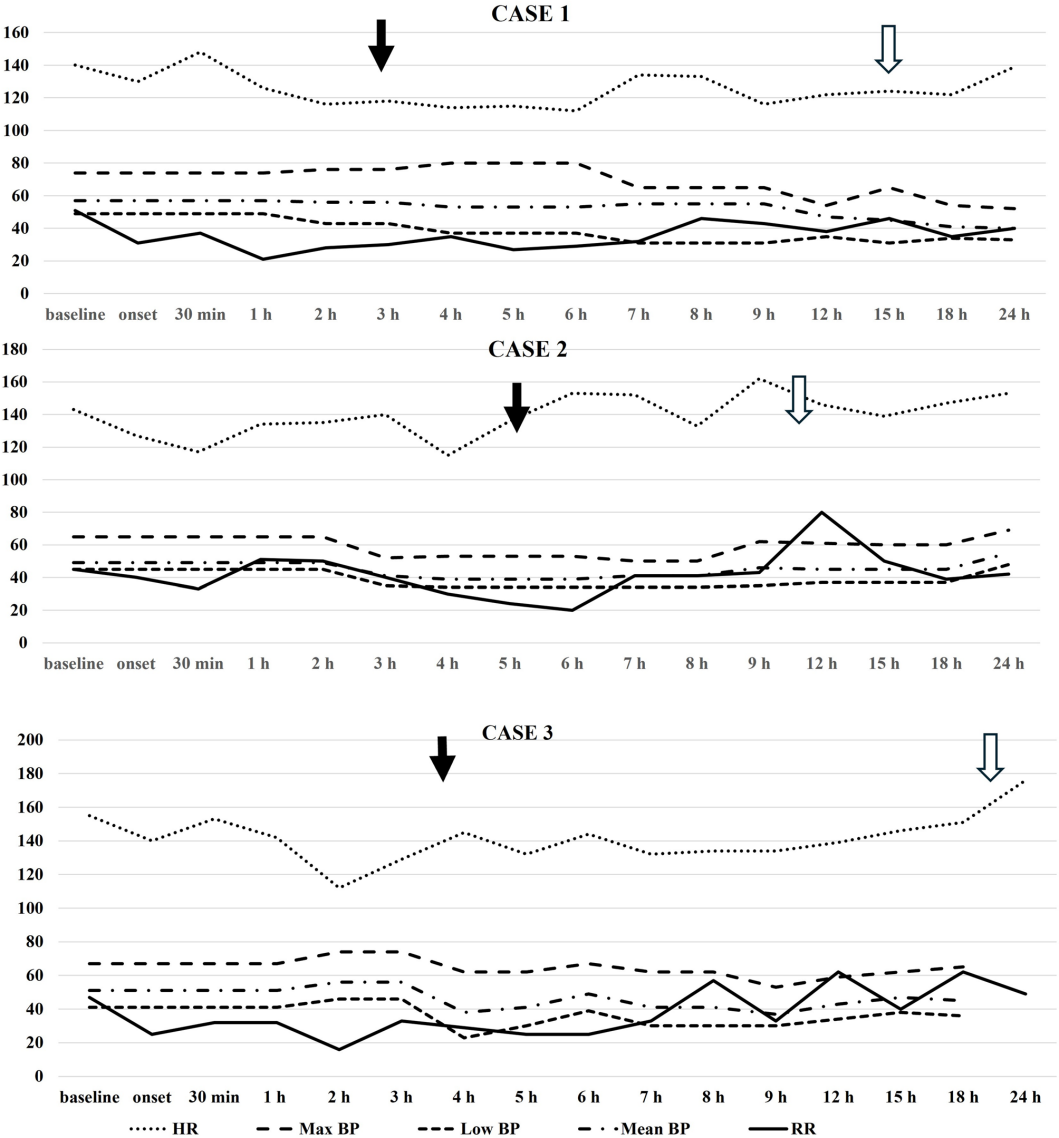
Heart rate (beats/min), systolic (Max BP, mmHg), diastolic (Low BP, mmHg) and mean (mmHg) oscillometric blood pressure, respiratory rate (RR, breaths/min) of the three infants at baseline, onset of symptoms and up to 24 h after onset. Full arrow indicates intubation, whereas empty arrow indicates extubation

The respiratory pattern normalized within a few hours; she was extubated about 15 h after onset of symptoms (Fig. 1). CFM tracing showed regular voltage and variability from 8 h after onset, baby’s behaviour normalized, glucose and lactates returned to normal, and all respiratory support was discontinued after 24 h. No similar episodes occurred subsequently.

### Case 2

A baby girl was born from a monochorionic-diamniotic twin pregnancy via C-section at 34 + 3 weeks of postmenstrual age, birth weight 1554 g. Apgar scores were of 8–9 at 1–5 min. CPAP was started at birth due to mild respiratory distress and discontinued on DOL4. Caffeine was started on DOL1 and continued because apnoeic episodes were still occurring.

She was regularly growing on enteral feeding by nasogastric tube and breathing in room air when on DOL16, at 36 + 4 weeks of postconceptional age, concomitantly to baby 1, on the same day at 9:00 a.m. she started to present similar symptoms including irritability, followed by poor reactivity, hypotonia, recurrent apnoeas, poor breathing effort with “gasping” appearance, hyperglycemia (glucose 307 mg/dL), increase in lactates (3 mmol/L), hypocapnia (16 mmHg).

Five hours after symptoms onset the baby was intubated. The respiratory pattern normalized within a few hours and then the baby was extubated 6 h later (Fig. 1). Treatments and laboratory data were similar to those of baby 1 (Table 1). Ammonia was normal. An electroencephalogram (EEG) started 1 h after symptoms onset showed significant depression of baseline electrical activity with suppression-burst pattern, which normalized spontaneously after a few hours. The baby’s behaviour rapidly returned to normal, and respiratory support was discontinued after 48 h (Fig. 1).

### Case 3

A baby boy was born from spontaneous vaginal delivery at 28 + 1 weeks of gestational age, birth weight 1296 g. Apgar scores were 7–8 at 1–5 min of life. CPAP was started at birth and continued with FiO2 21-25%, caffeine was started on DOL1. He received also 48 h of antibiotics and parental nutrition for 17 days.

He was stable in CPAP when on DOL20, at 30 + 6 weeks of postconceptional age, on the same day as the other two babies, at 09:30, he also started to appear hypotonic and less reactive with intermittent frequent episodes of apnoea, irregular breathing with a “gasping” appearance, occasional bradycardia in the absence of signs of circulatory compromise. Arterial blood exams showed hyperglycaemia (glucose 238 mg/dL), increased lactates (4.6 mmol/L), hypocapnia (26 mmHg). Clinical management and test results were similar to cases 1 and 2 (Table 1).

Due to apnoea he was intubated 4 h after onset of symptoms and supported with mechanical ventilation; the respiratory pattern progressively normalized, hypotonia improved and he was extubated after about 22 h (Fig. 1). Behaviour fully normalized the following day. He continued to require non-invasive respiratory support until 36 weeks’ gestational age.

Hospital risk management was immediately alerted. An intoxication was suspected since the three babies were located in adjacent cribs and had been cared for by the same nurse.

Urine, cerebrospinal fluid, blood and stool samples were then collected for toxicological analysis.

The review of the medication charts showed that all the three newborns had received at approximately 8 a.m. the same therapy by nasogastric tube, which included caffeine, iron and vitamin D.

**Fig. 2:**
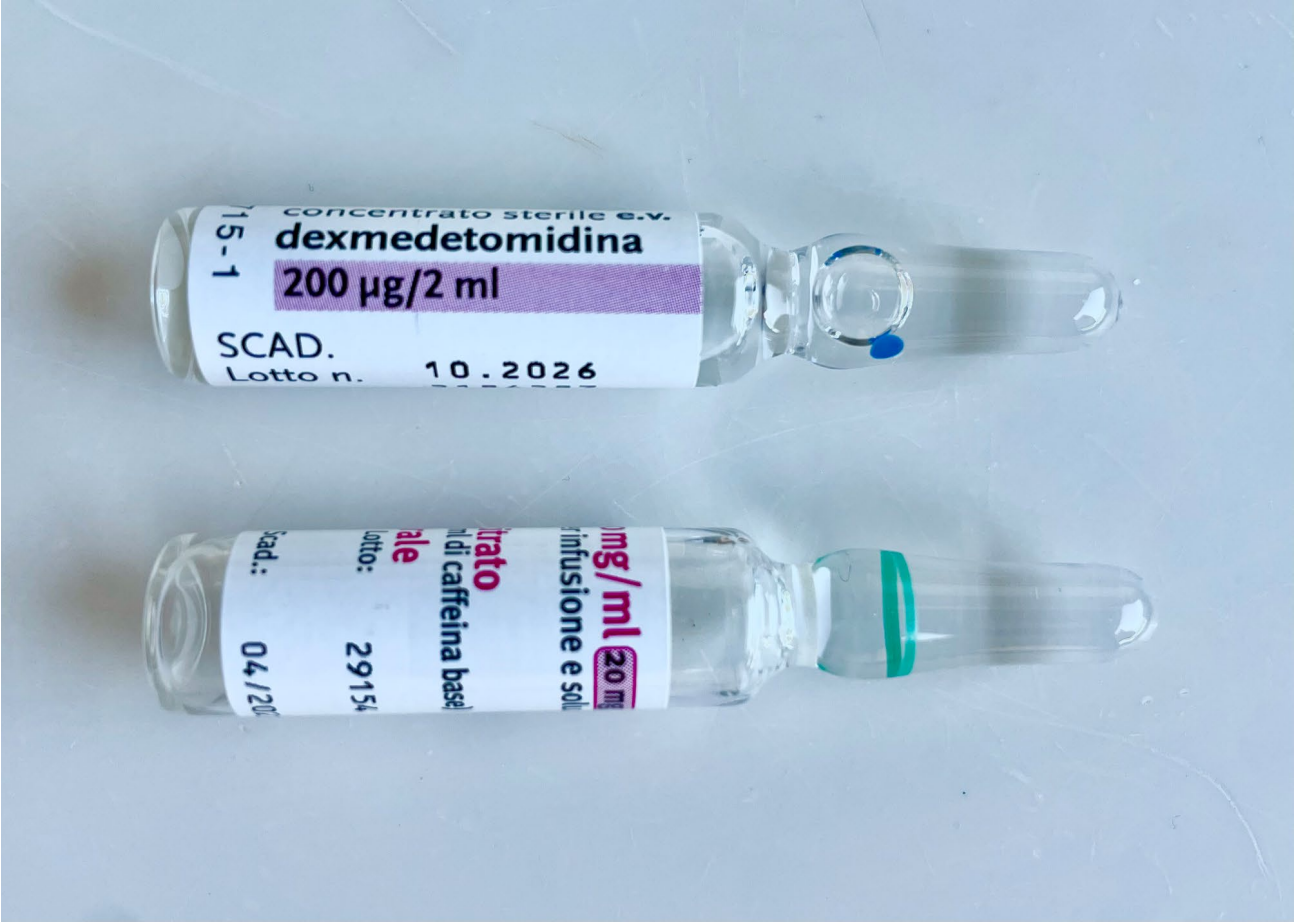
Dexmedetomidine and caffeine vials

Since another baby in the unit, shortly before 8 a.m., had received intranasal dexmedetomidine for comfort by using only half a vial, nursing personnel hypothesized that the one and half vial of dexmedetomidine remaining in the package might have been accidentally left on the medicine counter instead of positioned back inside medicine cupboard. For this reason, it might have been administered by mistake as caffeine to the three babies, half a vial each. Indeed, vials of dexmedetomidine and caffeine looked alike as both displayed pink and white colours and were of similar size and shape. (Fig. 2)

The review of literature confirmed the consistency between clinical signs and symptoms of the three babies and dexmedetomidine overdose.

On this basis, blood and urine samples, obtained approximately 10 h after onset of symptoms from the three babies, underwent the determination of dexmedetomidine. Analyses were performed by liquid chromatography coupled to triple quadrupole mass spectrometry (LC-QQQ MS) after protein precipitation for urine whereas blood was analyzed after liquid-phase extraction according to the procedure described by Partridge et al. [15]. Analyses were performed at Forensic Toxicology of the Dept. of Diagnostic and Public Health of the University of Verona. The analyses showed the presence of the drug in urine samples of all the three babies.

Families were then informed about the mistake and about the following management of the clinical cases. A report to Italian Medicines Agency (Agenzia Italiana del Farmaco, AIFA) was made. Clinical follow-up after discharge of the babies was regular.

The neonatal unit subsequently re-evaluated its drug management processes, implementing measures suitable for ensuring greater safety. In particular, dexmedetomidine and caffeine were stored in separate, distant locations within the medication cupboard. Access to the medication room was restricted to one nurse at a time, and a ‘Do Not Disturb’ sign was placed on the door during daily medication preparation [16]. The medicine counter was cleared, and nursing personnel implemented an educational initiative to emphasize the importance of repositioning drugs in the cupboard after use.

## Discussion and conclusions

We described dexmedetomidine overdose in three preterm newborns, following accidental enteral administration in the context of “look alike” medication error.

The symptoms of the three infants included initial irritability followed by hypotonia, hyporeactivity and a suppression-burst pattern on EEG and CFM tracings.

Sedative and anxiolytic dexmedetomidine activity is mediated by inhibition of central sympathetic output in locus coeruleus, with relative increase in inhibitory neurons activity, while its analgesic effect derives from a reduction in P substance release in spinal cord dorsal horns [2]. At high dose, deep brain inactivation may occur. Whereas, at lower doses, dexmedetomidine in children elicited an EEG pattern similar to that of sleep with modest increases in theta, alpha, and beta activity [17].

Prolonged sedation following intravenous dexmedetomidine accidental overdose in children was previously reported [9–13]; in one of these cases, irritability upon stimulation, as in our cases, occurred [11].

Our infants showed a peculiar “gasping” respiratory pattern with recurrent apnoea that eventually required intubation. Dexmedetomidine is usually deemed not to depress spontaneous respiration. However, preterm infants might be more susceptible to respiratory depression following deep sedation. Bradypnea was also reported in a 11 Kg child who received a rapid bolus infusion of 100 micrograms of dexmedetomidine [12]. Hypocapnia, we documented on initial blood gas analysis, seems inconsistent with the documented pattern of respiratory depression; we postulate that profound intermittent breaths during the “gasping” phase, maybe secondary to increased lactates, might have determined this finding.

Haemodynamic alterations were not striking in our babies. No significant bradycardia nor hyper/hypotension occurred, requesting intervention, although we observed a poor heart rate variability with steady lower values.

Dexmedetomidine has been associated with a biphasic effect on blood pressure, with hypertension secondary to peripheral alpha-2 receptors stimulation with vasoconstriction at high doses and hypotension and bradycardia due to central sympathetic inhibition at lower doses [18]. In paediatric overdose cases, hemodynamic instability was variably reported [9–13].

In the cases we described 2–4 h after enteral dexmedetomidine administration significant hyperglycaemia was detected; it was no longer present at subsequent controls carried out in the following hours. Alpha receptor inhibition by dexmedetomidine in pancreatic cells may inhibit insulin release and therefore cause hyperglycaemia [19]. In healthy children undergoing induction anaesthesia, blood glucose levels were elevated from baseline in a dose-dependent manner 15 min after dexmedetomidine intravenous bolus. No residual effect was identified at 30 min [20]. A delayed enteral dexmedetomidine absorption and metabolism in preterm infants may explain longer persistence of hyperglycaemia in our babies.

Conversely, decreased stress response and central sympathetic inhibition with reduced pancreatic beta stimulation together with fasting have been implicated in hypoglycaemia as reported after another case of paediatric overdose [13].

We estimated that, taking into consideration the volume of dexmedetomidine vials corresponding to the volume of prescribed doses of caffeine, the babies erroneously received approximately 45–50 micrograms/Kg of dexmedetomidine by nasogastric tube.

Onset of symptoms occurred around one hour after administration; babies required intubation after 3–5 h. All symptoms subsided after approximately 10–18 h.

In adults and children, oral bioavailability of dexmedetomidine is reported to be low (8–16%) with a high first passage metabolization by the liver [21]. Thereafter, extraction is prevalently urinary. In adults, peak plasmatic concentrations were retrieved 2–3 h after oral doses [21].

No studies are available on enteral dexmedetomidine pharmacokinetics or efficacy in newborns. However, in children given oral doses of 2–4 micrograms/Kg 30–60 min before procedures, a good sedative effect was achieved [22]. The delayed onset and prolonged effect we observed could be explained by hepatic and renal immaturity.

Our cases detail how dexmedetomidine overdose may appear in preterm newborns.

In particular, the “gasping” breathing pattern displayed by all babies was strikingly similar and easily recognizable, as detailed in video (see Supplementary Material, Video dex.MP4), and we hope it might be of help to suspect dexmedetomidine overdoses in other neonatal units.

Urine, cerebrospinal fluid, blood and stool samples were obtained from the babies; urine and blood were analysed by a specialized laboratory. This allowed us to identify with certainty dexmedetomidine overdose as the cause of symptoms. A good collaboration with hospital risk management and reference laboratory was important to assure this result.

We cannot state for certain that dexmedetomidine was administered in place of caffeine rather than of vitamin D or iron, as the action was not directly witnessed. However, this was the most likely hypothesis, given that both vitamin D and iron were in the form of suspensions and appeared very different from dexmedetomidine vials.

Labelling orientation and units of measurements of caffeine and dexmedetomidine vials were different. However, definition of look alike medication error is lacking [16] and sizes, shapes and colours of vials were indeed similar.

Although dexmedetomidine use is considered generally safe in newborns [3], careful storage and management should be implemented due to possible medication error that may lead to serious central nervous system and respiratory depression in preterm newborns.

In conclusion, oral dexmedetomidine overdose due to medication error is possible in newborns and should be suspected in case of clinical presentations like the one of the three reported cases. Measures should be implemented in neonatal units for a safe use of dexmedetomidine.

## Electronic supplementary material

Below is the link to the electronic supplementary material.

Supplementary Material 1: CARE-checklist_dex.pdf: pdf, CARE checklist

Supplementary Material 2: Video dex.MP4: MP4 file, video of clinical presentation of one of the babies described

## Data Availability

Data sharing is not applicable to this article as no datasets were generated or analysed during the current study.

## Abbreviations

NICU: Neonatal Intensive Care Unit
DOL: Day of Life
CFM: Cerebral Function Monitoring
EEG: Electroencephalogram
