# Supplementary material for: Accidental dexmedetomidine overdose in preterm newborns: a report of 3 cases
Source: Ital J Pediatr. 2025 Jul 6;51:211. doi: 10.1186/s13052-025-02060-1 (PMC12232678; doi:10.1186/s13052-025-02060-1)
Supplement: Supplementary file 1 — Supplementary Material 1: CARE-checklist_dex.pdf: pdf, CARE checklist [file 13052_2025_2060_MOESM1_ESM.pdf]

CARE case report guidelines

CARE Checklist of information to include when writing a case report

(cc) BY-NC-ND

Topic	Item	Checklist item description	Reported on Line
Title	1	The diagnosis or intervention of primary focus followed by the words "case report"	Lines 1-2
Key Words	2	2 to 5 key words that identify diagnoses or interventions in this case report, including "case report"	Line 42
Abstract (no references)	3a	Introduction: What is unique about this case and what does it add to the scientific literature?	Lines 28-30
	3b	Main symptoms and/or important clinical findings	Lines 31-35
	3c	The main diagnoses, therapeutic interventions, and outcomes	Lines 35-38
	3d	Conclusion—What is the main "take-away" lesson(s) from this case?	Lines 39-41
Introduction	4	One or two paragraphs summarizing why this case is unique (**may include references**)	Lines 58-69
Patient Information	5a	De-identified patient specific information.	Lines 77-79, 107-110, 124-127
	5b	Primary concerns and symptoms of the patient.	Lines 82-87, 111-120, 128-133
	5c	Medical, family, and psycho-social history including relevant genetic information	not applicable
	5d	Relevant past interventions with outcomes	Lines 79-81, 109-110, 125-127
Clinical Findings	6	Describe significant physical examination (PE) and important clinical findings.	Lines 82-87, 111-120, 128-133
Timeline	7	Historical and current information from this episode of care organized as a timeline	Figure 1
Diagnostic Assessment	8a	Diagnostic testing (such as PE, laboratory testing, imaging, surveys).	Table 1
	8b	Diagnostic challenges (such as access to testing, financial, or cultural)	Lines 145-155
	8c	Diagnosis (including other diagnoses considered)	Lines 156-157
	8d	Prognosis (such as staging in oncology) where applicable	Line 160
Therapeutic Intervention	9a	Types of therapeutic intervention (such as pharmacologic, surgical, preventive, self-care)	Lines 92-104, 116-118, 133-136
	9b	Administration of therapeutic intervention (such as dosage, strength, duration)	Lines 92-104, 116-118, 133-136
	9c	Changes in therapeutic intervention (with rationale)	not applicable
Follow-up and Outcomes	10a	Clinician and patient-assessed outcomes (if available)	Line 160
	10b	Important follow-up diagnostic and other test results	not applicable
	10c	Intervention adherence and tolerability (How was this assessed?)	not applicable
	10d	Adverse and unanticipated events	not applicable
Discussion	11a	A scientific discussion of the strengths AND limitations associated with this case report	Lines 218-228
	11b	Discussion of the relevant medical literature **with references**.	Lines 167-216
	11c	The scientific rationale for any conclusions (including assessment of possible causes)	Lines 167-216
	11d	The primary "take-away" lessons of this case report (without references) in a one paragraph conclusion	Lines 232-234
Patient Perspective	12	The patient should share their perspective in one to two paragraphs on the treatment(s) they received	not applicable
Informed Consent	13	Did the patient give informed consent? Please provide if requested	Yes ☒ No ☐
